# PVA-Based Nanofibers Containing Chitosan Modified with Graphene Oxide and Carbon Quantum Dot-Doped TiO_2_ Enhance Wound Healing in a Rat Model

**DOI:** 10.3390/jfb13040300

**Published:** 2022-12-15

**Authors:** Fatemeh Norouzi, Mehrab Pourmadadi, Fatemeh Yazdian, Keyvan Khoshmaram, Javad Mohammadnejad, Mohammad Hossein Sanati, Faraz Chogan, Abbas Rahdar, Francesco Baino

**Affiliations:** 1 Department of Biology, Nourdanesh Institute of Higher Education, Meymeh, Isfahan 8351765851, Iran; 2 Department of Life Science Engineering, Faculty of New Science and Technologies, University of Tehran, Tehran 1439957131, Iran; 3 Department of Physics, Faculty of science, University of Zabol, Zabol 538-98615, Iran; 4 Institute of Materials Physics and Engineering, Department of Applied Science and Technology, Politecnico di Torino, 10129 Torino, Italy

**Keywords:** chitosan, poly(vinyl alcohol), carbon quantum dot, titanium dioxide, graphene oxide, wound healing

## Abstract

Electrospun nanofibrous constructs based on nanoparticles and biopolymers have recently been used in tissue engineering because of their similarity to the extracellular matrix in nature. In this study, electrospun chitosan-carbon quantum dot-titanium dioxide-graphene oxide (CS-CQD-TiO_2_-GO) nanofibrous mats were synthesized for use as wound dressings by the electrospinning method. To increase the biodegradation rate and water resistance, the fabricated nanofibrous mats were cross-linked. SEM images showed a uniform and coherent structure of CS-CQD-TiO_2_-GO nanocomposites and CS-CQD-TiO_2_-GO electrospun nanofibers mats. FTIR analysis, XRD pattern, SEM mapping, and EDS spectrum demonstrate the accuracy of the synthesis as well as the elemental and chemical structure of the nanofibrous mat. The water contact angle indicated that the nanofibrous mat had a hydrophilic property, which is essential for controlling wound exudates. The tensile strength and elongation tests showed that the nanofibrous mat has suitable mechanical properties for wound dressing, including significant flexibility and strength. Interestingly, antimicrobial testing illustrated that the fabricated nanofibrous mat had antibacterial activity against Gram-negative and Gram-positive bacteria. Appropriate cell viability and cytocompatibility of treated mouse fibroblast NIH3T3 cells with the nanofibrous mat were determined using an MTT assay. The animal study results confirmed the proper potential of the nanofibrous mat in wound dressing applications.

## 1. Introduction

Wound healing issues and subsequent economic and health aspects involve millions of people all around the world [1]. Complex interactions between various cell types, elements of the extracellular matrix, and soluble factors are required for effective wound healing [2]. Therefore, the use of novel dressings that actively aid in wound healing has been taken into consideration [3]. Recent studies indicate that the use of nanoparticles (NP) in dressings plays an effective role in producing active dressings to accelerate wound healing [4]. Among efficient nanoparticles, TiO_2_ shows antibacterial activity against some Gram-positive and Gram-negative bacteria strains. Good proliferation of animal fibroblast cells exposed to compounds containing TiO_2_ also indicates that it is non-toxic [5]. However, TiO_2_ is a photo-catalyst nanoparticle and is activated under the influence of harmful UV irradiation and can only use up to 5% solar energy [6]. Additionally, the low quantum efficiency is a result of the low rate of electron transfers to oxygen. Therefore, it has been suggested that doping TiO_2_ with carbon-based NPs will improve its separation of electron-hole pairs and move its absorption to the visible portion of the spectrum [7,8]. Combining TiO_2_ with metal elements of carbon quantum dots increases the efficiency of nanoparticles [9]. CQDs as carbon-based NPs have multiple functional groups, such as carbonyl, hydroxyl, carboxyl, and amino groups, which increase the range of emission wavelengths and excitation [10]. This allows the CQDs to react easily with TiO_2_ and reduce the TiO_2_ bandgap energy to be activated in the visible light spectrum. The remarkable properties of carbon such as excellent dispersion, biocompatibility, low toxicity, good cell permeability, and easy functionalization with biomolecules have made CQDs applicable in drug delivery systems and wound-dressing [10,11,12]. The small size of CQDs makes them an attractive nanocarrier for drug delivery applications, and they have revealed negligible toxicity in animal studies [13]. Interestingly, recent studies have proven that the TiO_2_-CQD compound not only has antibacterial properties but is also a qualified option as medicine for wound healing [14]. On the other hand, some studies have suggested that combining graphene oxide (GO) with TiO_2_ increases the antibacterial power of TiO_2_ under solar energy. GO is a two-dimensional carbon-based nanostructure that has received much attention due to its potential of eliciting antibacterial effects, enhancing angiogenesis, improving cell proliferation, and facilitating rapid wound healing [15]. Furthermore, GO owns unique chemical and physical properties with diverse functional groups, high biocompatibility, and proper stability, which make it a favorable material for biomedical applications [16]. It has been demonstrated that incorporation of nanomaterials such as GO and reduced GO (rGO) with polymeric biomaterials can improve cell proliferation and blood vessel formation. Reducing GO minimizes the amount of oxygen as it makes GO unstable [17]. A recent work reported the angiogenic property of appropriate concentrates of GO and rGO through in vitro and in vivo angiogenesis assays by controlling reactive oxygen species (ROS) formation [18]. ROS can function as a signaling molecule in numerous growth factor-mediated physiological processes, including cell proliferation and wound healing [19]. However, the inherent aggregation characteristic of GO could result in dispersion restrictions that are strongly dependent on their surface interaction. To modify the GO surface, grafting hydrophobic nanoparticles could increase the layer gap and modify the GO behavior to a loose state while preserving its properties [20]. TiO_2_ is an acceptable option for GO dispersion decoration. Thus, we have used GO in polymeric nanocomposite to accelerate wound healing and enhance cell migration. TiO_2_-CQD-GO nanocomposites (NC) have the potential of being both antibacterial and non-toxic. However, the use of nanocomposites is much more effective in nanofibers mode since nanofibrous structures exhibit surprising features such as high surface area-to-volume ratio and great porosity [21]. The ability of nanofiber-based NC to mimic the diameter of elongated collagen fibrils in the natural extracellular matrix makes it an ideal scaffold for tissue regeneration. Nanofibrous scaffolds offer an alternative artificial extracellular matrix for cell adhesion and proliferation due to their microporosity, antibacterial activity, surface roughness, hydrophilicity, and high surface area [22,23]. For instance, PVA nanofibers were fabricated in order to facilitate wound healing by incorporating carbon nanotubes and Ag nanoparticles [24]. PVA/ZnO nanocomposite fibers were also utilized for wound healing, which resulted in accelerated reepithelization, increased angiogenesis, and better cell attachment [25].

This study used chitosan (CS) polymer as a substrate for TiO_2-_CQD-GO NPs to facilitate nanofiber fabrication. Chitosan is a biodegradable and biocompatible polymer that can imitate the structure and function of the natural extracellular matrix. It is one of the many options used to produce biomedical nanofibers [26,27]. The electrospinning technique was used as one of the best methods for the production of polymeric nanofibers [21]. Since CS is hardly electrospinnable, it needs to be blended with an electrospinnable polymer such as poly(vinyl alcohol) (PVA) [28]. The reason for the preference of PVA over other polymers (e.g., poly(ethylene oxide)) is its desirable interactions at the molecular level with CS [21,29,30]. The novelty of this research was the synthesis of the CS-GO-CQD-TiO_2_ NC, which has been used after electrospinning as an effective antibacterial pad to accelerate wound healing.

## 2. Material and Methods

### 2.1. Chemicals and Materials

RPMI-1640, nitric acid (HNO_3_), phosphoric acid (H_2_PO_4_), sulfuric acid (H_2_SO_4_), phosphate buffer saline (PBS), graphite flakes, KMnO_4_, fetal bovine serum (FBS), MTT (3- (4,5-dimethylthiazol-2-yl)-2,5 diphenyl tetrazolium bromide), H_2_O_2_, and DMSO (dimethyl sulfoxide) were brought from Sigma (St. Louis, MO, USA). Diethylenetriamine, anhydrous tetrahydrofuran, thionyl chloride (SOCl_2_), poly(vinyl alcohol) (PVA), and dodecyl amine were bought from Merck, Germany, and the NIH 3T3 fibroblast cells were obtained from ATCC (Virginia, US).

### 2.2. Synthesis of Nanoparticles

#### 2.2.1. CQD Synthesis by Hydrothermal Method

Initially, 2 g of diammonium hydrogen citrate was added to 75 g of water and stirred until completely dissolved. After that, the mixture was put to the hydrothermal autoclave, which was then heated to 150 °C for 18 h. Finally, CQD was acquired at a concentration of 26.66 mg·mL^−1^ [10,31,32].

#### 2.2.2. Synthesis of Graphene Oxide (GO)

There are various techniques for graphene production, including the Hummer method [15,33,34]. The initial step in the synthesis of GO monolayer is to add 1 g of graphite to 20 mL of 98% pure sulfuric acid on the stirrer within an ice bath to completely dissolve the graphite at a low temperature by a magnet within the sulfuric acid. After around 30 min, 3 g of potassium permanganate was progressively added to the solution, which had formed a green sludge. After roughly 30 min, 50 mL of distilled water was added dropwise. After 10 min, 100 mL of distilled water was added to the solution while swirling with a magnet. After 30 min, 35 mL of hydrogen peroxide was poured drop-by-drop, and the graphene oxide solution was removed from the heat stirrer after 24 h [35,36,37,38].

#### 2.2.3. Indirect CQD-TiO_2_ Synthesis Method

An amount of 10 mL of CQD synthesized in the previous step was brought to a concentration of 2 mg·mL^−1^. In a separate vessel, 10 mg of TiO_2_ was dissolved in 100 mL of distilled water. The two solutions were mixed for 1 h at 27 °C to homogenize thoroughly. Then, 0.2 g of sodium borate hydrate was poured into the solution as a reducing agent to react on the stirrer at room temperature for 2 h and release hydrogen gas. Finally, CQD-TiO_2_ was gained at a concentration of 1.4 mg·mL^−1^ [14].

#### 2.2.4. GO-CQD-TiO_2_ Synthesis Method

In total, 100 mg GO was added to the synthesized CQD-TiO_2_ to be completely homogeneous on the stirrer for 1 h. Then, 0.02 g of sodium borate hydrate was added and placed on a stirrer for 2 h to homogenize completely. Then, it was ultrasonicated for 10 min to dissolve the solution completely. Finally, a GO-CQD-TiO_2_ nanocomposite was obtained at a concentration of 6.4 mg·mL^−1^. The freeze-drying method was also used to powder the sample. The sample was centrifuged and then refrigerated at −20 °C until it became completely frozen and finally dried.

#### 2.2.5. CS-GO-CQD-TiO_2_ Synthesis Method

For the preparation of CS-GO-CQD-TiO_2_ NC, the spraying method was used. For this purpose, 750 µL of CS-GO-CQD-TiO_2_ NC solution was prepared with a concentration of 6.4 mg·mL^−1^. Then, pure glutaraldehyde (with a volume ratio of 0.05 glutaraldehyde/composite) in dark conditions was added to the solution to create cross-links and increase the strength of the composite. Finally, the resulting solution was placed on a stirrer at room temperature for 5 min to obtain a homogeneous solution.

### 2.3. CS-GO-CQD-TiO_2_ NC Electrospinning Process

In total, 0.5 g of PVA in 5 mL of DI water was dissolved on a stirrer under vigorous agitation for 1 h, and a homogeneous solution was obtained. The solution was then filled with 350 µL of GO-CQD-TiO_2_ NC solution and uniformly stirred for 30 min with a stirrer. With the use of a stirrer, 0.25 g of chitosan was dissolved in 0.6 mL of acetic acid and added to the pre-made nanocomposite solution and, finally, the solution was prepared for electrospinning. The NC solution was fed into a 5 mL syringe with a 22-gauge needle. Then, the syringe was placed in the appropriate position, as well as the attachment of two positive and negative electrodes to the aluminum foil of the collector plate to initiate the flow in the polymer solution. As a result, the electrospinning process began. Then, a syringe with a capacity of 5 mL was filled with the solution and located in the electrospinning setup. A constant flow of solution with a 0.5 mL·h^−1^ rate, and constant conditions, including 15 kV voltage, 15 cm distance between the collector and tip of the needle, and 28 °C temperature, were applied. The electrospun sample was placed in the open air for 24 h to ensure the evaporation of any solvent.

### 2.4. Cross-Link Process of Nanofibers

The nanofibrous mats were cross-linked to enhance the mechanical characteristics. Additionally, cross-linking increases nanofiber water resistance ability. Cross-links originate from interactions between the polar hydroxyl groups of PVA, NPs, and chitosan’s amine groups and the aldehyde groups of glutaraldehyde [39]. In this paper, glutaraldehyde was used to make cross-linking in the nanofibers. The sample was exposed to glutaraldehyde for 24 h in an oven desiccator at 50 °C. Because of the toxicity of glutaraldehyde, the nanofibers were washed with 1% glycine, and distilled water was used to remove unreacted glutaraldehyde. The samples were then placed in the oven to dry completely [40].

### 2.5. Morphological, Chemical, and Microstructural Characterization

Synthesized NPs and nanofibers were characterized by zeta potential, Dynamic Light Scattering (DLS), and Scanning Electron Microscope (SEM) techniques. The dry nanofibers were used in order to conduct SEM analysis. Furthermore, the nanocomposites were well dispersed in order to detect the size, zeta potential, and morphology through the mentioned techniques. Other characteristics were defined by Energy Dispersive X-ray Spectroscopy (EDS), SEM mapping, Fourier Transform Infrared Spectroscopy (FTIR), and X-ray Diffraction (XRD).

### 2.6. Static Water Contact Angles (WCA)

WCA was measured using the sessile-drop method. A specially sized drop (e.g., four microliters in ASTM D7334 standard) was applied to the sample [41]. The contact angle test was performed statically on the surface of the nanofibers using a Jikan Gac-10 device. A distilled water drop of approximately 4 μL microliters was placed on the surface of nanofibrous mats measuring 2 × 1 cm^2^ at room temperature. A maximum of three drops was applied to each sample. Angles were then obtained using Image J: Drop-Analysis software and the mean of three measurements was reported for each sample. These angles indicate the wettability of the solid surface by the liquid [10,42].

### 2.7. Degradability of CS-GO-CQD-TiO_2_

To investigate the biodegradability of CS-GO-CQD-TiO_2_, the 1 × 1 cm^2^ fragment of the NC was dried, weighed, and placed in a Petri dish containing 10 mL of PBS with pH = 4.7 and stored at 37 °C for 30 min. Then, it was kept in the oven for 7, 14, 28, 42, 56, 72, and 96 h. Finally, the sample was incubated at room temperature for 3 days to dry completely, and then its dry weight was measured using a digital scale. Degradability was obtained from the following formula [40]:

(1) $$\mathrm{Biodegradability}\;\left(\%\right)=\frac{\mathrm{Primary}\;\mathrm{dry}\;\mathrm{weight}-\mathrm{Sec}\mathrm{ondary}\;\mathrm{dry}\;\mathrm{weight}}{\;\mathrm{primary}\;\mathrm{dry}\;\mathrm{weight}}\times 100\;$$

### 2.8. Antibacterial Properties of CS-GO-CQD-TiO_2_ Nanofibers

The antibacterial activity of the nanocomposites was evaluated by using two indicator bacteria. The characteristics of the bacteria used are listed in Table 1.

The liquid MIC of CS-GO-CQD-TiO_2_ was obtained as reported in a previous article [14]. The sterile paper disk was coated with a minimal inhibitory concentration of CS-GO-CQD-TiO_2_ at a rate of 100 g·mL^−1^. The disc was heated for 1 h at 45 °C to dry. *E. coli* and *S. aureus* were cultured uniformly with sterile swabs on Neutron Agarculture medium. Then, the CS-GO-CQD-TiO_2_ disks were placed on the surface of each bacterium at a certain distance from the antibiotic disk. Furthermore, to test the growth inhibition zone made by CS-GO-CQD-TiO_2_ NC, gentamicin for *S. aureus* and tetracycline for *E. coli* were employed as controls. Then, using a ruler to measure the growth inhibition zone’s diameter all around the discs, the data were evaluated using the standard CLSI table.

### 2.9. Cytotoxicity Induced by CS-GO-CQD-TiO_2_ Nanofibers

CS-GO-CQD-TiO_2_ NC was first extracted. Thus, for each 5 mg, the NC, 1 mL of culture medium was added. After incubation, the samples were added to mouse fibroblast cells at different concentrations of 0.01, 0.06, 0.1 µg·mL^−1^, and 0.6 at 3 and 5 day intervals. The culture medium was used as a control. In total, 10^6^ NIH3T3 fibroblast mouse cells were cultured in a 96-well plate and then incubated for 24 h at 37 °C to evaluate cytotoxicity by the MTT method. Extracts from each sample were added to the respective wells on a plate, and the NIH3T3 cells were exposed to them for 24 h. Then, 100 mL of colorless PRMI and 10 mL of a 12 mM MTT solution were added to each well after the growth medium had been removed. The supernatant medium was removed after 4 h, and 100 L of DMSO was added to each well. Finally, the amount of OD was calculated at 570 nm. The cytotoxicity test of samples was repeated three times, and the OD was measured in triplicate.

### 2.10. Tensile Strength and Elongation Tests

The mechanical characteristics were evaluated by assessing elongation-at-break and tensile strength with a Texture Analyzer in accordance with the Standard Test Procedure for Tensile Properties (ASTM) standard method [43]. These tests are carried out under circumstances where strips of preconditioned CS-GO-CQD-TiO2 composite that are 1.0 cm (W) × 5.0 cm (L) in size are fitted between the machine’s grips with an initial grip gap of 50 mm. The sample was pulled from one side until broken at a cross-head speed of 2.0 mm/min. To obtain the average values, each test trial contained 5 replicate measurements. The tensile and elongation tests were shown by MPa (%) and percentage, respectively [3].

### 2.11. Cell Migration Inhibition Study by Scratch Wound Assay

A scratch wound assay was used to evaluate the effect of CS-GO-CQD-TiO_2_ on cell migration. This test was performed with minor modifications similar to the technique used by Malmir et al. [14]. First, NIH3T3 fibroblast mouse cells were seeded in 24-well plates, and then they were given the night to attach to the plate bottom. After 2 h of starvation in 0.1% FBS medium, a sterile 10 µL pipette tip was used to puncture vertical wounds at the cell surface. Cells were treated with CS-GO-CQD-TiO_2_ and then incubated for 24 h at 37 °C. There was a 100 µm solution in each well. As a negative control, cells that were treated with the medium were used. At the beginning of the incubations and again after 24 h, the damaged cells were imaged using a Nikon Eclipse Te-2000-U microscope (4X magnification). Using Image J software, the scratch area’s surface A was measured. For each well, the results were calculated using the following formula: 100 × (A_t24_/A_t0_). A Student’s t-test was used in the statistical analysis, which was done using the Sigma Plot software.

### 2.12. Animal Study

By injecting 10 mg kg^−1^ (body weight) of ketamine and xylazine intraperitoneally while the rats were under anesthesia, the dorsal area of their bodies was shaved from the scapula to the ileum, leaving a shear wound that measured 20 mm × 20 mm. They were then split into 4 groups, non-treated rats, phenytoin-treated rats, PVA-treated rats, and CS-CQD-TiO_2_-GO electrospun fiber-treated rats. The first day following wound induction, treatment was begun; topically applied polymers were used, and the wounds were treated. On the seventh and fourteenth days, all groups’ wounds were inspected and photographed. Finally, the affected region was cleaned, captured on camera, and estimated with ImageJ software (version 1.44 p). The following formula was used to calculate the wound healing rate [9]:

(2) $$\mathrm{Wound}\;\mathrm{healing}\;\left(\%\right)=\frac{W_{0}-W_{1}\;}{W_{0}}\times 100$$

where W_0_ and W_1_ refers to wound area at initial and interval time points, respectively.

A one-way ANOVA test analyzed the data obtained from the wound area measurement.

### 2.13. Histological Analysis

An identically positioned skin sample was taken from each mouse at the end of the experiment. The tissue samples were embedded in paraffin, fixed in 4% formaldehyde, and cut into slices that were about 4 mm thick. Hematoxylin, eosin (H&E), and TRM were used to stain the sections (Trichrome staining). Their morphological modifications were then assessed.

### 2.14. Statistical Analysis

The experiments in this study were repeated at least three times for each analysis, and the related data were expressed as mean ± standard deviation. ANOVA tests were performed based on groups using GraphPad Prism software. A *p*-value less than 0.05 was considered statistically significant.

## 3. Results and Discussion

### 3.1. Particle Size and Zeta Potential of CQD-TiO_2_

**Figure 1:**
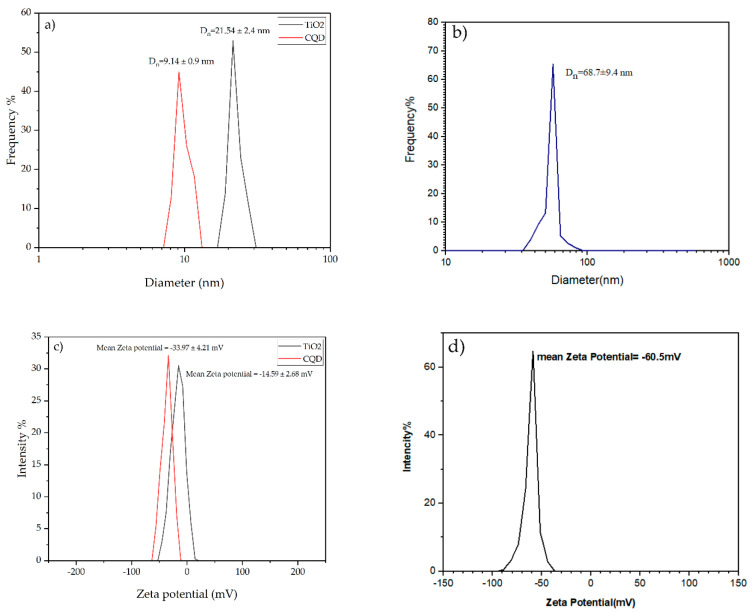
Dynamic light scattering of (**a**) CQD and TiO_2_, (**b**) CQD-TiO_2_; zeta potential of (**c**) CQD and TiO_2_, (**d**) CQD- TiO_2._

Evaluation of DLS test results for pure CQD, TiO_2_, and CQD-TiO_2_ nanoparticles in Figure 1a,b shows the presence of nanoparticles with an average size of 9.14, 21.54, and 68.7 nm, respectively. CQDs are quantum NPs that may have a diameter of less than 10 nm [44]. Adding other NPs to CQDs leads to an increase in dynamic light scattering [10]. Therefore, the 59 nm diameter of CQD-TiO_2_ NPs might be a preliminary indication that TiO_2_ NPs are linked to CQDs. Figure 1c,d shows the zeta potential of pure CQD, TiO_2_, and CQD-TiO_2_. Particles with a zeta potential greater than 30 mV or less than −30 mV are stable. The reported zeta potential of −60.5 mV for CQD-TiO_2_ indicates its good stability [45].

### 3.2. Morphology of CS-GO-CQD-TiO_2_

**Figure 2:**
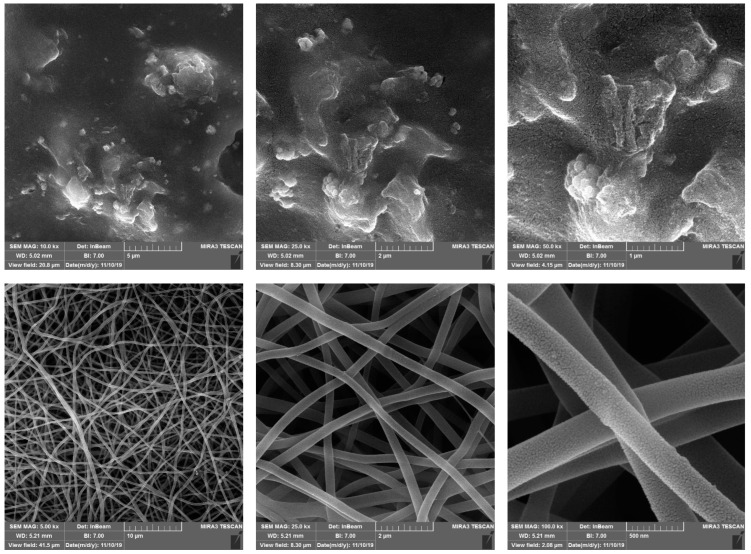
SEM morphology of (**top**) CS-CQD-TiO_2_-GO NC and (**bottom**) PVA and CS-CQD-TiO_2_-GO NC electrospun nanofibrous mat.

The SEM images of the CS-GO-CQD-TiO_2_, electrospun PVA, and CS-GO-CQD-TiO_2_ NC with a magnification of 10,000 times are displayed in Figure 2. In Figure 2 (top), a coherent structure with some roughness is seen. This compact structure indicates that the GO, CQD, CS, and TiO_2_ NPs were well connected. The roughness seen in Figure 2 (top) is due to the presence of large chitosan crystals. The addition of CS to NC has minimized the free volumes and increased the cohesion of the microstructure of the NC [46]. The results of increased condensation and roughness due to the presence of chitosan in the NC is consistent with the results of Yaowen Liu et al. [47]. In this regard, the SEM images of the research of Ahmed et al. showed that the GO NPs are well dispersed in the synthesized polymer, and filling the porosity of the polymer is effective in its final performance [48]. Another study suggested that, according to SEM images, the presence of CS-GO NPs in the polymer increased the roughness and height of the polymer [49]. Figure 2 (bottom) shows the electrospun CS-GO-CQD-TiO_2_ containing the PVA. The coherent surface and the obtained nanofibers illustrated the high compatibility of PVA with the NC and a compact structure lacking phase separation [50]. The absence of air bubbles, cracks, or pores further supports the excellent compatibility of the CS with PVA, as shown by Tripathi et al. [50].

### 3.3. FTIR Analysis of Materials and Nanocomposites

**Figure 3:**
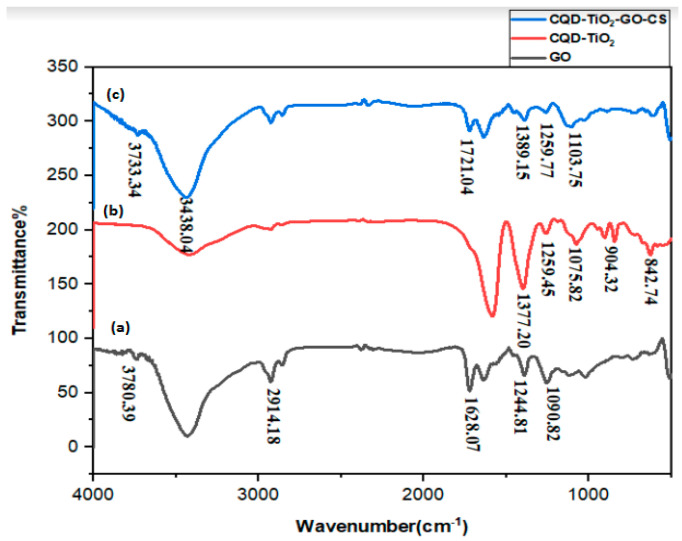
FTIR spectrum of GO (**a**), CQD-TiO_2_ (**b**), CQD-TiO_2_-GO-CS (**c**).

FTIR analysis of GO, CQD-TiO_2_, and CS-GO-CQD-TiO_2_ is illustrated in Figure 3a–c. Comparatively, in Figure 3a, GO plates have different peaks in the range of 1105 cm^−1^ (C-O), 1200 cm^−1^ (C-O-C), and 1600 cm^−1^ (C=O), and are broadband in the range of 3000 to 3700 cm^−1^. Oxidizing agents have effectively oxidized the graphite as evidenced by the presence of oxygenated functional groups [51]. Furthermore, the detected bands of GO (Figure 3a) at 1628 and 1550 cm^−1^ correspond to the carboxyl group bending vibration and the C=C skeletal vibration, respectively [52]. In Figure 3b, the C-OH tensile vibration peak is located between 1000 and 1300 cm^−1^, and the C-O-C bond exhibits vibration at 1163 cm^−1^. The absence of peaks in 1350–1460 cm^−1^, which is related to CQD, indicates that CQD-TiO_2_ NPs are well-synthesized, and these results are similar to the recent papers [14]. The shifts of the peaks in Figure 3c indicate that the NPs in the CS-GO-CQD-TiO_2_ are well-bonded together. According to relevant studies, chitosan has two firm peaks in 1534 cm^−1^ and 1640 cm^−^1, which are related to amide II (N-H bending variations) and amide I (C=O stretching), respectively [53,54]. The C-O stretching in chitosan may be responsible for the absorption peak at 1090 cm^−1^. The C-H stretch vibrations are thought to be responsible for the peak at about 2900 cm^−1^ [55].

### 3.4. FTIR Analysis of Nanofibers

**Figure 4:**
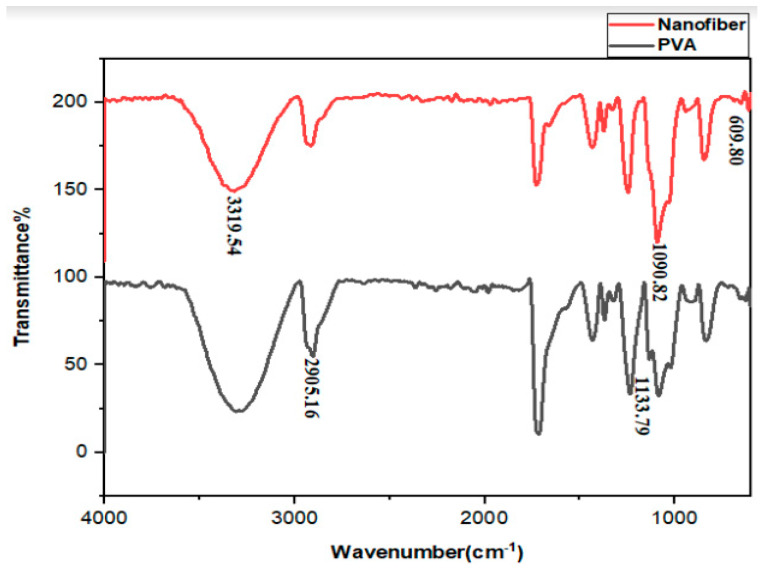
FTIR spectrum of PVA and CS-CQD-TiO_2_-GO electrospun nanofibrous mat. The red graph represents the CS-CQD-TiO_2_-GO nanofiber, and the black chart shows the PVA FTIR spectrum.

Figure 4 reports the FTIR diagram of electrospun CQD-TiO_2_-GO-CS and PVA. To facilitate CS-GO-CQD-TiO_2_ electrospinning, the NC has been combined with PVA [21]. Diana Isela and colleagues also combined chitosan with PVA in their research to facilitate the electrospun process of chitosan and nanofiber preparation [56]. Figure 4 indicates the FTIR of PVA. C-H tensile bond vibrations at 2916 cm^−1^ and the C-O bond at 1372 cm^−1^ belong to PVA. These results are in line with the results of Hassan Adeli et al. [40]. Characteristic absorption peaks of the components were shown by the nanofibrous PVA-CS-GO-CQD-TiO_2_ mat. It is possible to attribute the O-H peak shift at the composite nanofibrous mat to the creation of hydrogen bonds between the hydroxyl or amino groups in CS and the hydroxyl groups in PVA in GO-CQD-TiO2 and PVA in CS [57].

### 3.5. XRD Analysis

**Figure 5:**
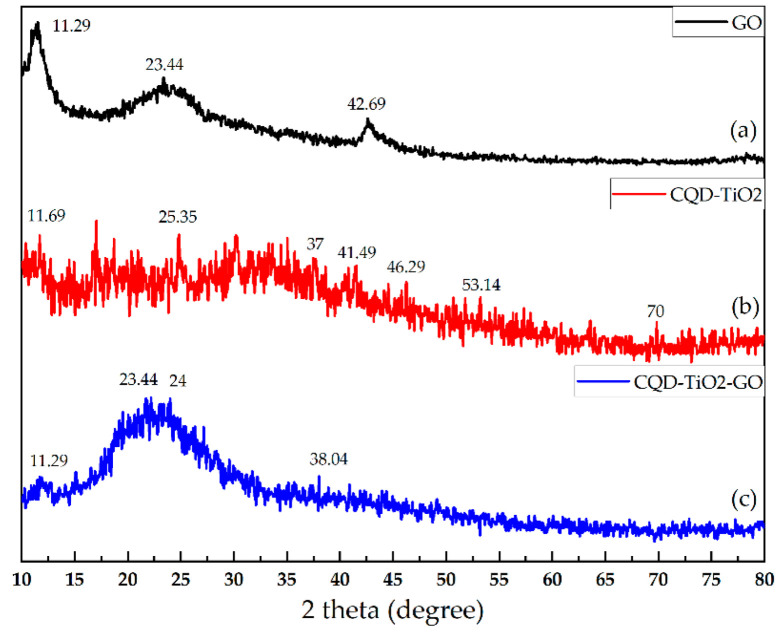
XRD pattern of GO (**a**), CQD-TiO_2_ (**b**), GO-CQD-TiO_2_ (**c**).

Figure 5a, b, and c display the pattern of XRD from GO, CQD-TiO_2_, and CQD-TiO_2_-GO, respectively. According to previously published papers, graphene oxide has a peak in the 2θ ~ 10° region with a base distance of d_001_ = 6.33 Å, which is used for characterization [58]. As shown in Figure 5a, the peak obtained from GO in the range of 2θ = 11.29° and 23.44° is a characteristic of oxygen containing functional groups of GO. Another 2θ peak at 42.69° can be seen, which is attributed to the turbostratic band of disordered carbon components. These results are in accordance with Stengl et al. [59]. The XRD pattern of CQD-TiO_2_ NPs in Figure 5b shows reflections at 25.19°, 37.77°, and 47.96°, indicative of CQD-TiO_2_, and these results are consistent with our previous paper [14]. As illustrated in Figure 3c, the intensity of peaks was decreased, which can be caused by the amorphous behavior of synthesized nanocomposites and incorporation of CS; however, broad and weaker bonds of GO, TiO_2_, and CQD can be seen in the profile. The weak peak seen at 2θ ~ 10–12° in Figure 5c indicates the presence of GO, which is caused by the small size of GO [59]. Studies have also shown that peaks around 25.4° are related to TiO_2_ [60]. Therefore, it can be concluded that the peak created at 2θ = 24° is the result of the overlap of CQD, TiO_2_, and GO NPs in the CQD-TiO_2_-GO NC.

### 3.6. SEM Mapping and EDS Analysis

**Figure 6:**
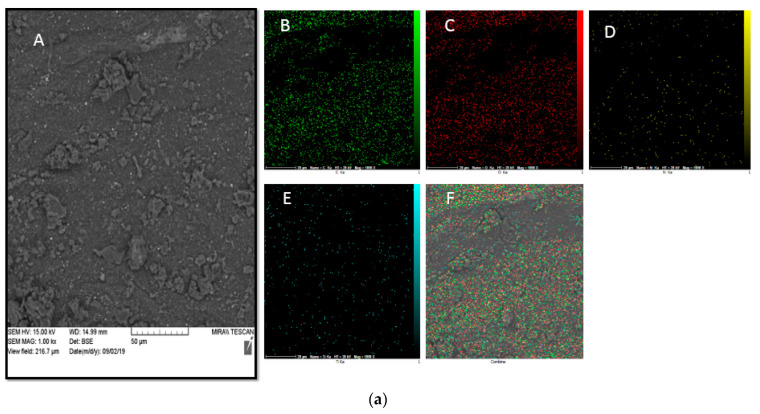

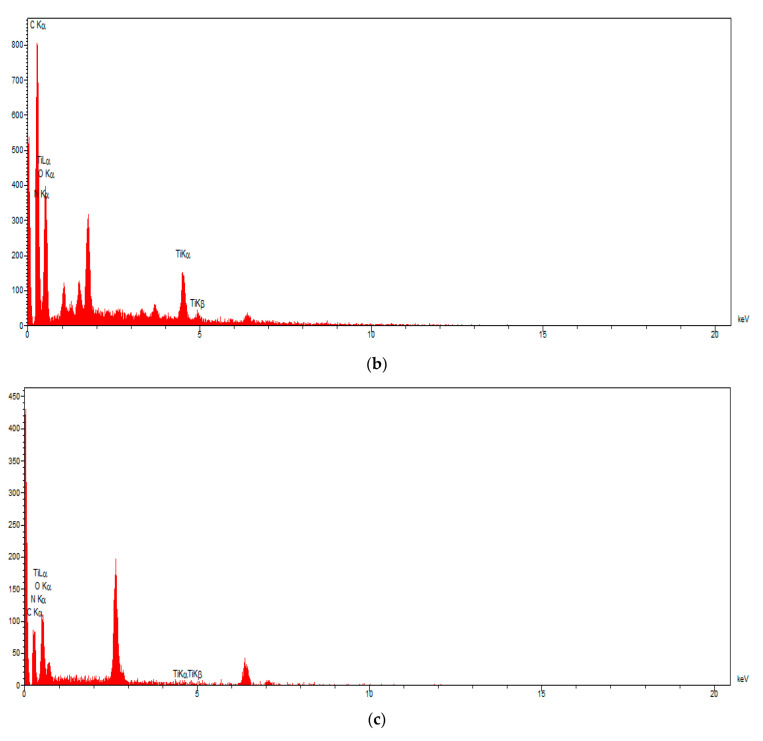

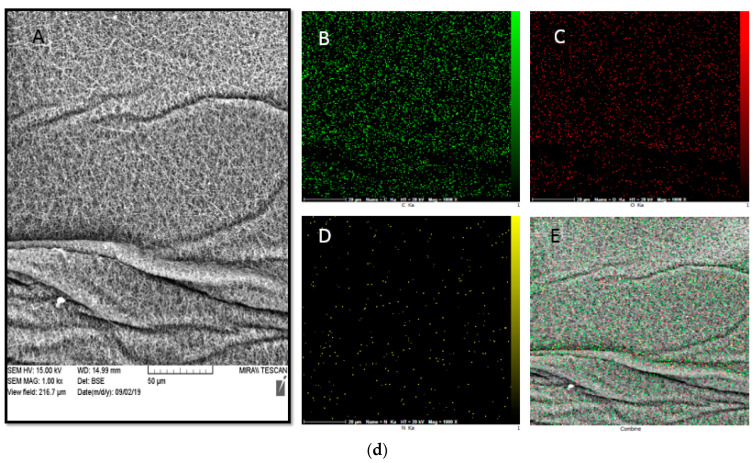
(**a**) Surface structure and elemental analysis of CS-GO-CQD-TiO_2_ NC: (A) SEM morphology of CS-GO-CQD-TiO_2_ NC; Images (B–E) represent carbon, oxygen, titanium, and nitrogen elements; Image (F) shows a combination of C, O, N and Ti elements. (**b**) EDS spectrum of CS-CQD-TiO_2_-GO NC. (**c**) EDS spectrum of CS-CQD-TiO_2_-GO electrospun nanofibrous mat. (**d**) Surface Structure and Elemental Analysis of PVA: (A) SEM morphology of PVA; Images (B–D) represent carbon, oxygen, and nitrogen elements; Image (E) shows a combination of C, O, and N elements.

Figure 6a,b show SEM mapping and the EDS analysis of CS-CQD-TiO_2_-GO electrospun nanofibrous mats. SEM mapping for PVA was also carried out (Figure 6c) for the purpose of comparison. Figure 6a shows the uniform bead-free structure of the electrospun nanofibers. Similarly, Koosha et al. observed a uniform bead-free morphology for electrospun nanofibers of CS/PVA by SEM mapping and the EDS analysis [23]. The EDS spectrum of the electrospun nanofibers in Figure 6b confirmed the presence of C, O, N, and Ti elements related to CS, CQD, TiO_2_, and GO NPs. The presence of the Ti elements in comparison with Figure 6a,c indicates the significant distribution of the NPs in the PVA. Furthermore, the characteristic peaks of C, O, N, and Ti elements in Figure 6b were detected, showing that the NPs were uniformly distributed inside the nanofibers. The elemental compositions of the NC and nanofibers are reported in Table 2.

### 3.7. The Hydrophilicity of Different Electrospun Nanofibres

**Figure 7:**
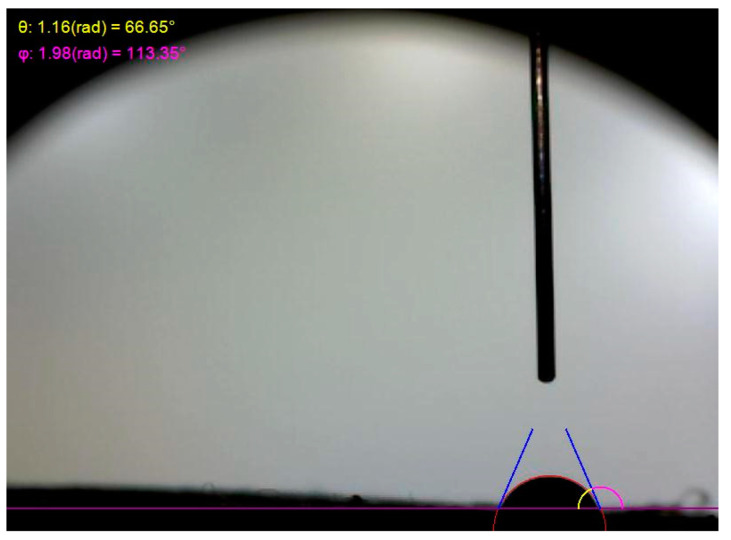
CS-CQD-TiO_2_-GO electrospun nanofibrous mat hydrophilicity. The angle θ (WCA) is equal to 66.65°.

The secretions that are made during an ulcer to the surface of the wound create a favorable environment for the development of bacteria [61]. One of the critical variables in making a viable wound dressing is its capacity to assimilate water. The surface hydrophilicity of CS-CQD-TiO_2_-GO electrospun nanofibrous was evaluated using dynamic water contact angle (WCA) measurements. The WCA of CS-CQD-TiO_2_-GO electrospun nanofibrous mats is depicted in Figure 7. The nanofibrous mat’s WCA value was 66.65°. As mentioned in the articles, WCA over 90° means that the polymer surface is hydrophobic [62]. Therefore, it can be concluded that CS-CQD-TiO_2_-GO nanofibrous mats are hydrophilic. Notably, two essential factors affect the WCA value, namely the physical hydrophobicity of the material itself and the membrane surface roughness [63]. For example, in a previous paper, it has been shown that a hydrophobic polymer-based composite can be made by bonding hydrophobic substances to the surface of GO [64].

### 3.8. Weight Loss

**Figure 8:**
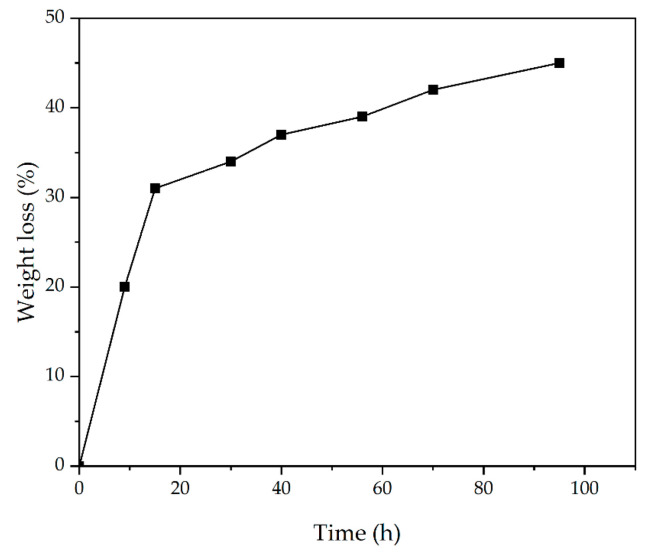
Weight loss of CS-CQD-TiO_2_-GO electrospun nanofiber. At first, biodegradability has increased, and it seems that this slope is almost fixed over time.

The produced nanofibrous mats should have an adequate degradation rate to provide suitable dressing during the wound healing process [40]. Figure 8 shows the biodegradation rate of the mats immersed in the PBS solution. According to Figure 8, it is evident that in the early hours of the sample placement in PBS, the process of degradation of the composite with a steeper slope was performed. However, over time, the process has declined. Similar to these results, previous studies have shown that chitosan mats have a lower degradation rate than PVA, also revealing that chemical cross-linking between the amine groups of glutaraldehyde and chitosan causes slower depolymerization and less degradation [65,66].

### 3.9. Mechanical Properties

**Figure 9:**
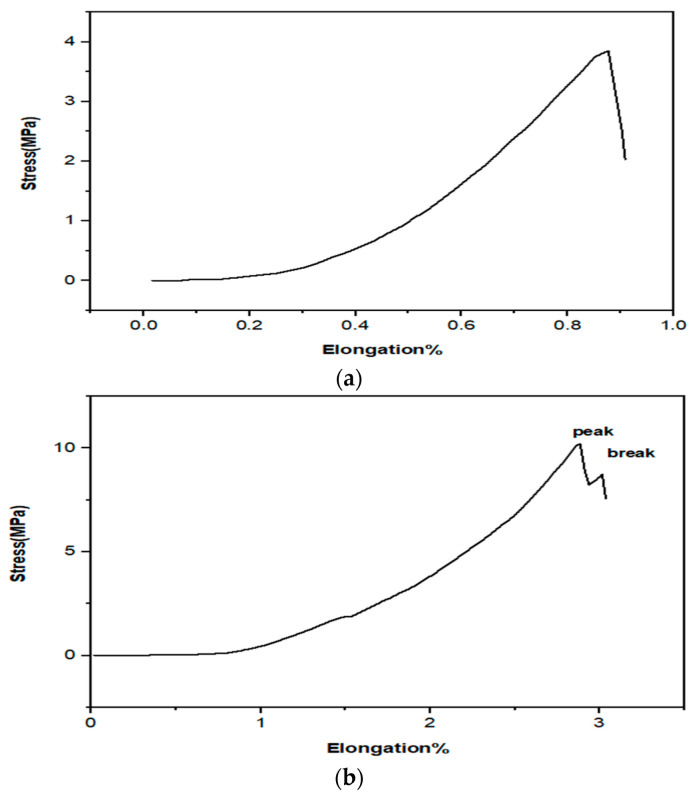
(**a**) Stress-elongation diagram in terms of pure bacterial cellulose. (**b**) Stress-elongation diagram in terms of bacterial cellulose/CS-CQDs-TiO_2_-GO electrospun nanofiber.

The nanofibrous mats should be able to provide suitable mechanical characteristics and flexibility in the electrospun state for improved clinical operation in wound dressing application [67]. The resulting nanofibrous mats’ elongation at break and tensile strength are shown in Figure 9b. The mechanical properties were suitable for the application in wound dressings and tissue engineering. The human skin elongation varies between 17% and 207% for elongation at break and between 1 and 32 MPa for tensile strength; however, because of the diversity of human skin, these values also rely on other variables such as age, genetic background, and skin color [68]. Figure 9b shows that the electrospun CS-CQD-TiO_2_-GO NC with PVA might offer greater tensile strength and elongation at break than the CS-CQD-TiO_2_-GO NC (Figure 9a). Notably, the synthesized mats in this research had very suitable mechanical properties, making the nanofiber flexible during wound coverage. Their tensile strength due to the presence of TiO_2_ ceramic NPs is higher than that reported by Agrawal et al. for PVA/chitosan nanofibers (1.33–6.15 MPa) [65].

### 3.10. Cell Viability

**Figure 10:**
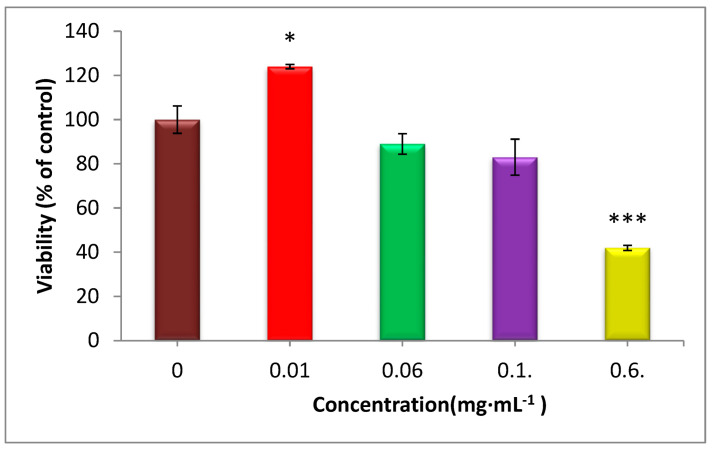
The cytotoxic effects of CQD-TiO_2_-GO-Cs NC on survival and proliferation of NIH3T3 fibroblast cells. The lowest toxicity was observed at the concentration of 0.01 μg·mL^−1^ (significant difference in comparison to control group: * *p* < 0.05 *), and the highest toxicity was at the concentration of 0.6 μg·mL^−1^ of the NC (significant difference in comparison to control group: *** *p* < 0.001).

It was anticipated that the perfect wound dressing must be significantly biocompatible [69]. An MTT assay was used to detect the cytotoxicity effects of the CS-CQD-TiO_2_-GO electrospun nanofibrous on Matson mouse fibroblast NIH3T3 cells. Figure 10 displays the results of this experiment at 0.06, 0.01, 0.6, and 0.1 µg·mL^−1^ concentrations based on nanocomposites compared to the control after 24 h. The NC solution was toxic at concentrations of 0.6 and 0.1 μg·mL^−1^, which after one day caused growth inhibition in fibroblast cells. The NC at the concentration of 0.06 μg·mL^−1^ had almost no toxic effects (cell viability about 90%) on fibroblast NIH3T3 cells. However, after 24 h, the concentration of 0.1 g·mL^−1^ promoted the development and proliferation of fibroblast cells, proving the safety of the produced NC at this concentration rather than having toxic effects on the cells. The results demonstrated that CS-CQD-TiO_2_-GO nanofibrous mats are biocompatible and suitable for tissue engineering applications. The results are consistent with the studies of Hassan Adeli et al. on electrospun PVA-CS nanofibers [40].

### 3.11. Antibacterial Test

**Figure 11:**
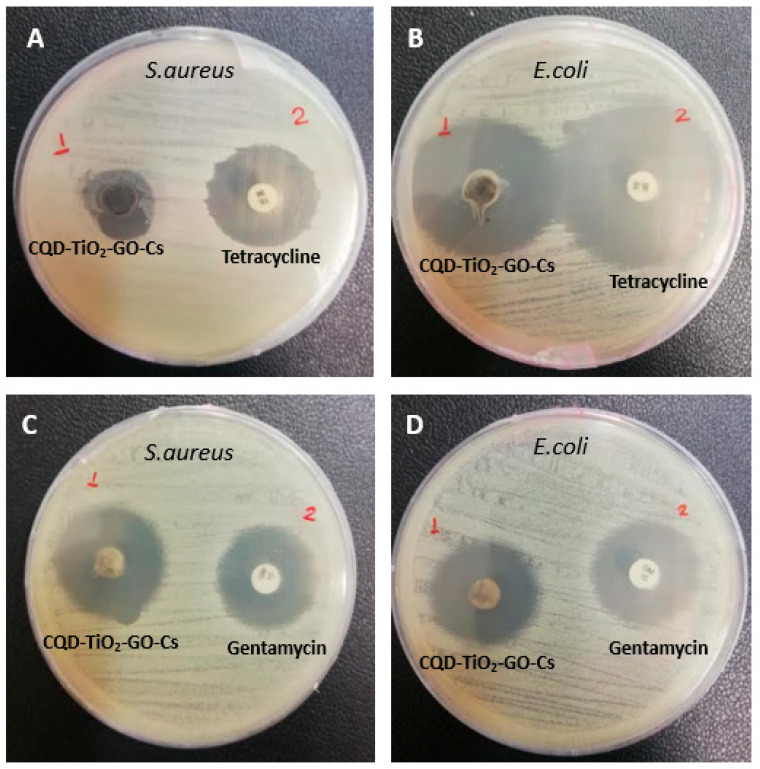
Comparison of antibacterial properties of CQD-TiO_2_-GO-Cs NC with tetracycline and gentamicin antibiotics. (**A**) *S. aureus*-containing plate with NC and tetracycline discs. (**B**) *E. coli*-containing plate with NC and tetracycline discs. (**C**) Plate containing *S. aureus* bacteria with NC and gentamicin discs. (**D**) plate containing *E. coli* with NC and gentamicin discs. Depending on the picture, the CQD-TiO_2_-GO-Cs NC has a more substantial effect on *E. coli* bacteria.

Previous studies have shown that *Staphylococcus aureus* bacteria are the main cause of wound infections [70]. Moreover, *Escherichia coli* bacteria are the common cause of burn wound infection [71]. Therefore, these bacteria can reduce the wound healing process [72]. The MIC concentrations of CQD-TiO_2_-GO-Cs NC were 0.2 mg·mL^−1^ for *Staphylococcus aureus* and 0.1 mg·mL^−1^ for *Escherichia coli*. The paper disc was impregnated with the NC at the above concentrations. Gentamicin and tetracycline discs were used as controls. The results in Figure 11A showed that the average halo diameter around the NC disk for *S. aureus* was 12 mm, and the halo diameter around the tetracycline antibiotic disk was 21 mm on average. As shown in Figure 11C, the inhibition zone of *S. aureus* around the sample and gentamicin antibiotic disk was approximately equal. In addition, the mean halo diameter around the NC disk in Figure 11B was 27 mm for *E. coli*, whereas the mean halo diameter around the tetracycline antibiotic disk was 29 mm. The gentamicin antibiotic and the NC disk’s inhibition zones created in Figure 11D were approximately equal. The antibacterial activity was more effective toward the Gram-negative *E. coli* than Gram-positive *S. aureus* bacteria, presumably because of their different cell wall structures [70].

### 3.12. Inhibition of Cell Migration and Invasion

**Figure 12:**
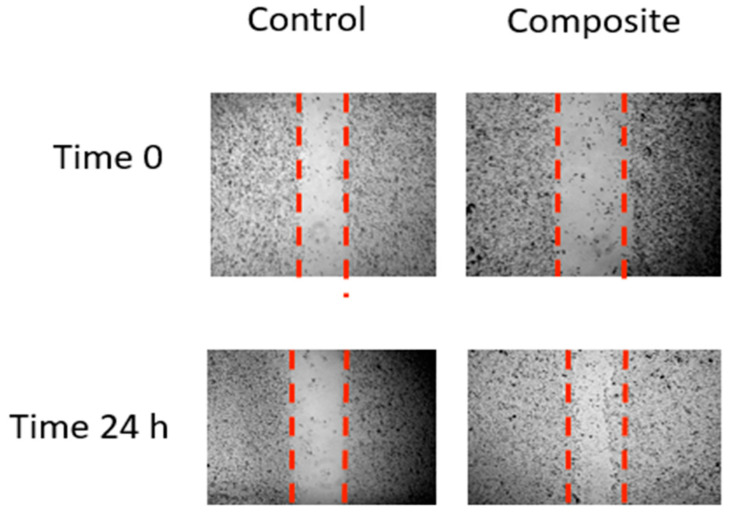
In vitro scratch test to measure the impact of the CQD-TiO_2_-GO-Cs NC on the migration of NIH3T3 fibroblast mouse cells. Then, at 0 and 24 h after scraping, cells were examined with an inverted microscope (4X magnification). The areas without cells are indicated by dotted lines.

A scratch wound assay was carried out to determine whether the treatment of CS-CQD-TiO_2_-GO influenced cell migration and invasion. For the controlled cells, considerable cell migration was seen after 24 h, as illustrated in Figure 12. However, after being treated with CS-CQD-TiO_2_-GO, injured NIH3T3 fibroblast mouse cell monolayers swiftly healed the gap over the course of 24 h, just like in the control group.

### 3.13. Animal Study

**Figure 13:**
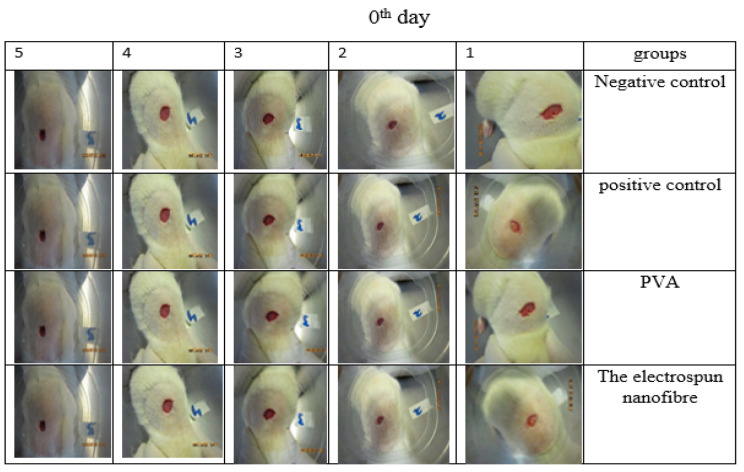
The rate of wound healing of 5 different mice treated in different groups on the first day. Wounds of about 1 cm × 1 cm were created in the skin of all mice, and mice were treated in different groups.

**Figure 14:**
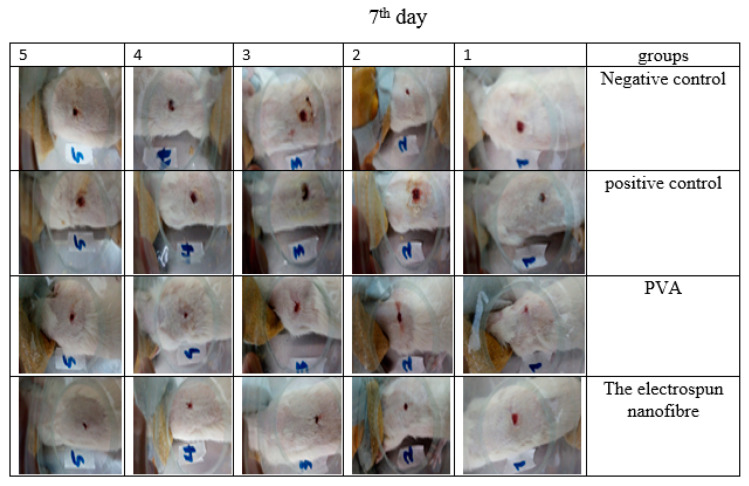
The rate of wound healing of 5 different mice treated in different groups on the 7th day. The highest rate of wound healing is related to the CS-CQD-TiO_2_-GO electrospun nanofiber treated group.

**Figure 15:**
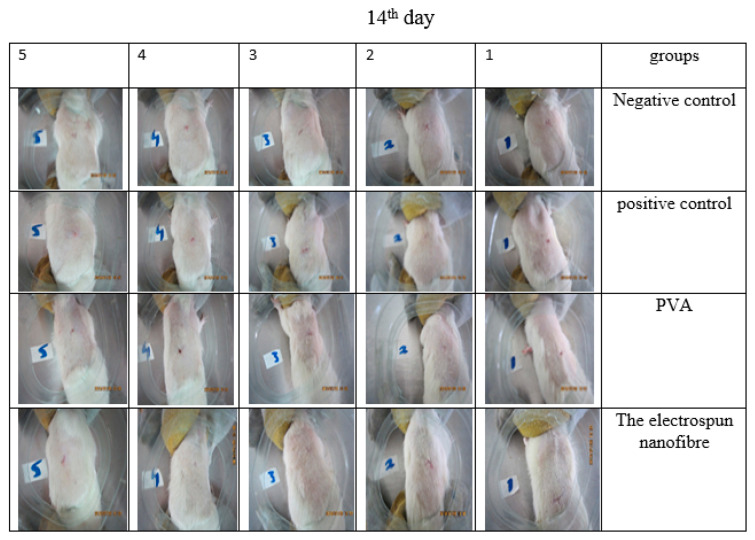
The rate of wound healing of 5 different mice treated in different groups on 14th day. As seen in the picture, the wounds of nanofibrous mat-treated mice have been completely healed.

**Figure 16:**
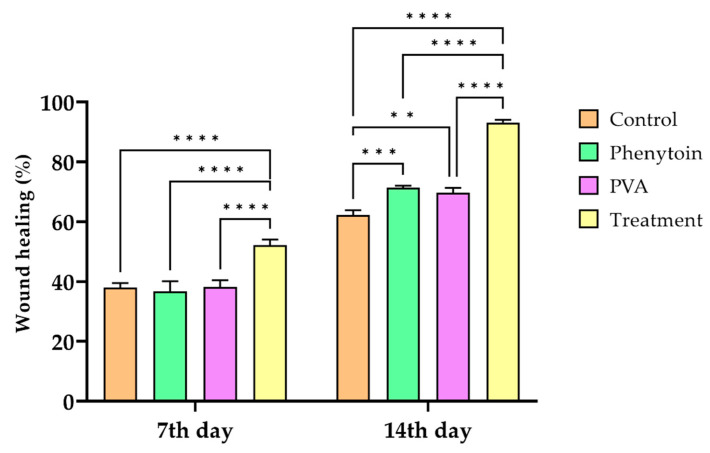
Graph of wound healing in the experimental groups after 7 and 14 days. According to the graph, the highest rate of wound healing was in the group treated with CS-CQD-TiO_2_-GO electrospun nanofibrous mats after 14 days. The treatment group had significant difference in comparison to other three groups at 14^th^ day (**** p < 0.0001); ** and *** reveal the difference between groups at *p* < 0.01 and *p* < 0.001, respectively.

Figure 13, Figure 14 and Figure 15 display the percentage of improved wound healing over the course of the 14-day study in all animal groups that received treatment. On the fourteenth day after receiving CS-CQD-TiO_2_-GO treatment, the wound area was significantly reduced in mice. The CS-CQD-TiO_2_-GO electrospun nanofibrous mats group demonstrated the best wound healing effect on the 7th day with a 52.102% improvement in comparison to other groups. As shown in Figure 16, the wound healing of the phenytoin group was compared with other groups at both 7th and 14th day. The wound area of phenytoin was comparable with the positive and negative groups and did not show any significant difference, which might be due to normal wound healing function [73]. On the 14th day, the CS-CQD-TiO_2_-GO electrospun nanofibrous mats group showed a 93.13% wound healing effect compared with both positive and control groups and phenytoin (*p* < 0.0001) (Table 3). Motealleh et al.’s research outcomes are similar to these results [74]. Furthermore, the nanofibrous mats showed good collagen organization on mice skin, and inflammatory responses were not observed. Graph of Figure 16 shows the wound healing contraction rate measured on the first, 7th, and 14th days. The results illustrate that the highest rate of wound healing was in the group treated with nanofibrous mats after 14 days. Similar to this study, in a paper by Marulasiddeshwara et al., it was demonstrated that CS-TiO_2_, CS, and curcumin NPs have synergistic effects, and TiO_2_-CS-curcumin NC results in the wound healing of treated mice after 14 days [75].

### 3.14. Histology

**Figure 17:**
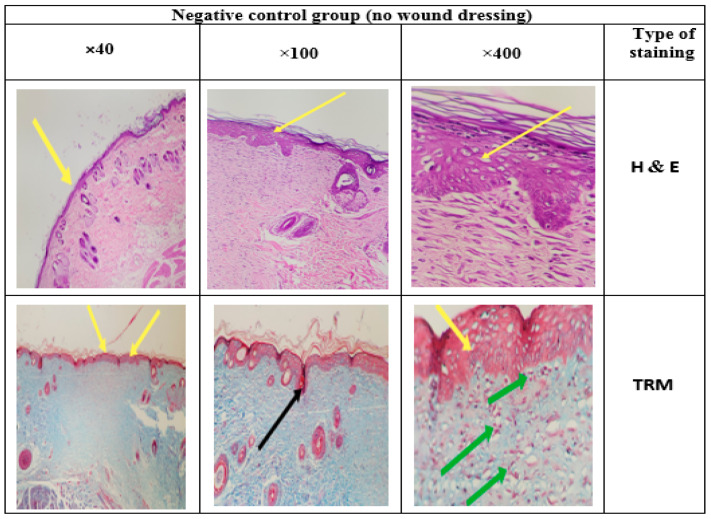
Microscopic imaging of skin tissue sections stained by two types of H&E and TRM dyes. The photo shows improved skin tissue 14 days after treatment in the non-wound dressing group. Yellow arrows indicate the formation of an epidermal layer in the tissue repaired with phenytoin, and also the green arrows indicate the penetration of inflammatory cells into the repaired tissue. Black arrow indicates dead cells in the tissue.

**Figure 18:**
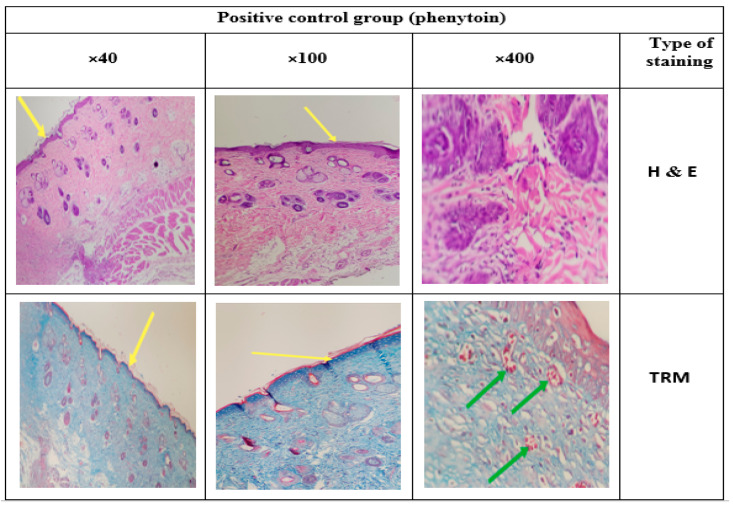
Microscopic imaging of improved tissue incisions 14 days after treatment in the positive control group (phenytoin). The yellow arrows indicate the formation of an epidermal layer in the repaired tissue that is treated with phenytoin, and the green arrows indicate the penetration of inflammatory cells into the repaired tissue.

**Figure 19:**
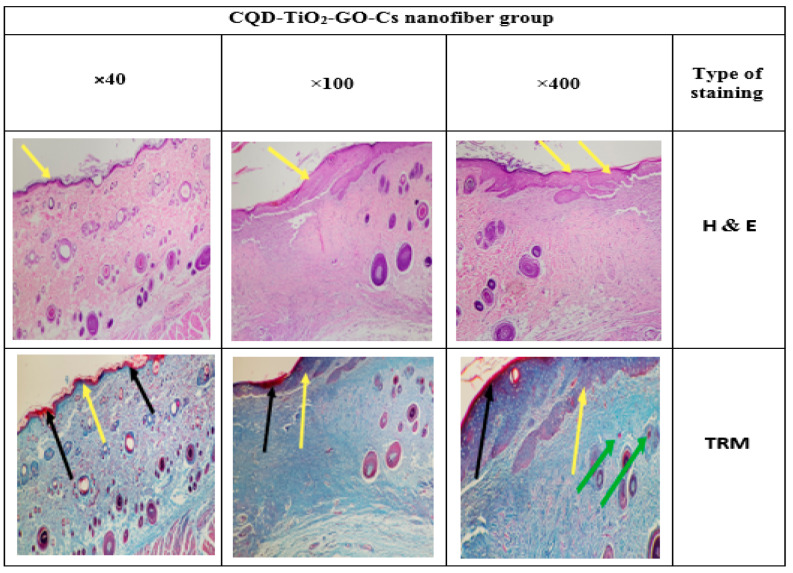
Microscopic imaging of improved tissue incisions 14 days after treatment with CQD-TiO_2_-GO-Cs nanofiber. Yellow arrows indicate the formation of an epidermal layer in the restored tissue treated with CQD-TiO_2_-GO-Cs nanofiber, and black arrows indicate dead cells in the tissue. Additionally, the green arrows indicate the penetration of inflammatory cells into the repaired tissue.

**Figure 20:**
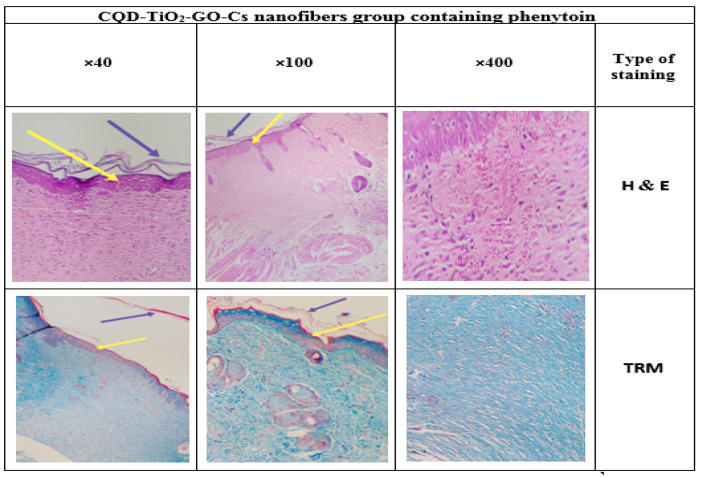
Microscopic imaging of improved tissue incisions 14 days after treatment with CQD-TiO_2_-GO-Cs nanofiber containing phenytoin. The yellow arrows indicate the formation of an epidermal layer in the repaired tissue along with the CQD-TiO_2_-GO-Cs nanofiber containing phenytoin. The purple arrows indicate the formation of creatine in the tissue.

Different epidermis layers can be well identified in the histological images in Figure 17, Figure 18, Figure 19 and Figure 20. To evaluate the effects of the CS-CQD-TiO_2_-GO nanofiber on the skin of injured mice, the wound skin tissue was examined in four groups after 14 days of treatment. The histology of all samples was examined by staining with H&E and TRM dyes. A similar method was performed by Bolle et al. [76] and Murphy et al. [77]. The negative control group included samples not receiving any treatment (Figure 17). The positive control group included the group treated with phenytoin (Figure 18). Figure 19 shows the group treated with the CS-CQD-TiO_2_-GO nanofiber and the last group treated with the CS-CQD-TiO_2_-GO nanofiber containing phenytoin (Figure 20). According to the results obtained from microscopic imaging of the four treatment groups and their comparison with each other, the wound healing stages in the CS-CQD-TiO_2_-GO nanofiber group containing phenytoin were more comprehensive than in the other three groups. This group also had an inflammatory response within 14 days after wound healing. It gradually decreased in this group, and dead cells in the tissue were much less seen than in the other groups, and creatine formation was observed on the fourteenth day.

## 4. Conclusions

In this study, novel electrospun nanofibrous CS-CQD-TiO_2_-GO mats were fabricated for wound healing application. FTIR analysis, XRD pattern, and SEM morphology indicated proper incorporation of NPs into composite and bead-free and uniform structures of the electrospun nanofibrous mats. Furthermore, the EDS spectrum of nanofibers confirmed the presence of related elements to CS, CQD, TiO_2_, and GO. The size and zeta potential of CQD-TiO_2_ were 68.7 nm and −60.5 mV, respectively. The mechanical properties were also investigated and the presence of the ceramic phases played a key role in increasing the tensile strength from around 3.8 to 9.0 MPa. Furthermore, the mats exhibited effective activities to inhibit bacterial growth of both Gram-positive and Gram-negative bacteria and showed proper cytocompatibility. The results of in vivo experiments in mice indicated that the CS-CQD-TiO_2_-GO composite resulted in better reepithelization and wound healing compared to the control group.

## Figures and Tables

**Table 1:** Characteristics of the bacteria used in antibacterial testing.

Bacterium Name	Standard Number	Gram Reaction
*Staphylococcus aureus*	PTCC1431	positive
*Escherichia coli O157:H7*	PTCC700728	negative

**Table 2:** Elemental composition table of NC and CS-CQD-TiO_2_-GO electrospun nanofiber.

NC		CS-CQD-TiO_2_-GO Electrospun Nanofiber	
Element	wt.%	Element	wt.%
C	44.21	C	37.75
N	6.42	N	7.43
O	49.37	O	54.52
Ti	-	Ti	0.31

**Table 3:** Wound healing percentage in experimental groups.

Groups		
	7th Day	14th Day
Control	38.025 ± 1.484	62.175 ± 1.583
Phenytoin	36.675 ± 3.435	71.34 ± 0.669 ^a^
PVA	38.163 ± 2.257	69.659 ± 1.597 ^a,e^
CS-CQD-TiO_2_-GO nanofibrous mats	52.102 ± 1.861 ^c^	93.137 ± 0.885 ^b,d^

Values are presented as mean ± standard deviation, ^a^ significantly different from control, *p* < 0.01, ^b^ significantly different from control, *p* < 0.001, ^c^ significantly different from PVA, *p* < 0.05, ^d^ significantly different from Phenytoin, *p* < 0.001, ^e^ significantly different from CS-CQD-TiO_2_-GO electrospun nanofibrous mats, *p* < 0.001.

## Data Availability

All data are included in the article.
